# Work re-entry and functioning in people with major depression: a longitudinal study of supported employment participants

**DOI:** 10.1186/s12888-025-06826-z

**Published:** 2025-04-18

**Authors:** Juliane Bergdolt, Stella Hubert, Julia Schreiter, Sarah Jenderny, Thomas Beblo, Martin Driessen, Ingmar Steinhart, Lorenz B. Dehn

**Affiliations:** 1 Universitätsklinik für Psychiatrie und Psychotherapie, Evangelisches Klinikum Bethel gGmbH, Universitätsklinikum OWL, Universität Bielefeld, Remterweg 69-71, 33617 Bielefeld, Germany; 2 Institut für Sozialpsychiatrie Mecklenburg-Vorpommern e.V, Universität Greifswald, Greifswald, Germany; 3 Department of Psychology, University of Bielefeld, Universitätsstraße 25, 33615 Bielefeld, Germany

**Keywords:** Functioning, Depression, Supported employment, Return to work, Disability, Vocational rehabilitation

## Abstract

**Background:**

People with major depression are affected by impaired daily functioning and non-participation in the workforce. At the same time, employment has been associated with improved mental health outcomes. The aim of this study was to examine the relationship between work re-entry and subsequent functioning and depressive symptoms.

**Methods:**

The present observational study included a sample of 129 participants of a supported employment intervention project in Germany diagnosed with depressive disorders. Participants were assessed before the start of the intervention (baseline) and after two years (follow-up). Functioning and depressive symptom severity were measured using the World Health Organization Disability Assessment Schedule 2 (WHODAS 2.0) and the Beck’s Depression Inventory II (BDI-II). After multiple imputation, linear regression analyses were conducted to analyze the relationship between work re-entry and follow-up functioning and symptom severity, controlling for baseline scores and age.

**Results:**

Work re-entry was significantly associated with better overall functioning (*p* =.002), cognitive functioning (*p* =.001) and community participation (*p* =.002), adjusted for baseline scores and age. A significant interaction effect (*p* =.001) suggested that the association between work re-entry and overall functioning at follow-up was stronger in older participants. After adjustment for baseline functioning and age, work re-entry was not significantly associated with self-care, social interaction and household responsibilities, while associations with mobility (*p* =.072) and symptom severity (*p* =.054) were marginally nonsignificant.

**Conclusions:**

The results support the association between work re-entry and lower disability in people with depression who participated in supported employment. Certain functional domains, especially cognition and participation, may be more closely associated with becoming re-employed. The association between work-re-entry and overall functioning may be stronger in older individuals.

**Trial registration:**

The data used for this study were collected as part of a clinical trial called “IPS-ZIB” which was prospectively registered with the German register for clinical trials on 12/14/2020 (DRKS; ID: DRKS00023521).

**Supplementary Information:**

The online version contains supplementary material available at 10.1186/s12888-025-06826-z.

## Introduction

Major depressive disorder (MDD) is one of the most common mental disorders and a major contributor to global burden of disease [1, 2]. It is characterized by the main symptoms depressed mood and diminished interest or pleasure in activities, and additional cognitive, behavioral or neurovegetative symptoms [3]. Importantly, MDD is also associated with significant functional impairment in various activities and areas of life [3]. The International Classification of Functioning, Disability and Health (ICF) [4], defines functioning and disability on three levels: (1) body functions/body structures (i.e. physiological functioning of the body), (2) activities and capacities (i.e. what someone is able to do), and (3) participation (i.e. involvement in life domains). According to the ICF’s biopsychosocial model, functioning and disability are dimensional constructs that result from the interaction of health conditions and contextual (personal and environmental) factors [4].

Because functioning is such a broad concept, both symptomatic and functional remission are important parts of recovery from depression. There is an increasing focus on functional recovery that relates to the ICF “activities and participation” section, i.e. restored ability to do or participate in certain activities [5, 6, 7, 8]. Patients themselves have also reported that returning to a usual level of daily functioning is one of the most important parts of recovery [9]. Although research has shown that improvement in depressive symptoms generally correlates with improvement in functioning [10, 11, 12], functioning is seen as a partly distinct concept that represents an own part of recovery [13, 14, 15]. For example, a recent systematic review concluded that even people in remission from MDD still had impaired functioning compared to healthy controls [16].

According to the ICF definition, functional impairment can be present in many different domains. One domain that is highly important at an individual and societal level is the ability to work in a competitive job, because work is a major part of life for many people. People with MDD not only struggle with reduced ability to work [17, 18], but are also more likely to be unemployed, on sick leave or apply for early retirement [19, 20, 21, 22, 23]. Thus, people with MDD are particularly affected by non-participation in the workforce, which is even more important given the assumed negative mental health consequences of unemployment [24, 25, 26, 27, 28].

At the same time, there is evidence that employment can be beneficial for mental health and specifically for depressive symptoms [29, 30, 31]. Transitions from unemployment to employment have been associated with improved mental health and quality of life in the general population [24, 32, 33, 34]. In addition, there is evidence that return to employment is associated with a variety of improved mental health outcomes in people with mental disorders. In particular, studies have identified an improved mental health status [35, 36, 37, 38], improved quality of life [39], self-esteem, mastery, physical health and happiness [38], as well as fewer psychiatric hospitalizations [39, 40, 41] and service use [42]. However, there may be concerns about competitive employment for people with mental disorders in terms of their readiness to work or potential harm that work may cause to their health. In fact, there is evidence that under adverse conditions, work can contribute to worsening mental health. For example, work-related stress may contribute to depressive symptoms and sickness absence due to mental disorders [43, 44, 45, 46]. Nevertheless, returning to work is a common and important goal reported by people with mental disorders [47, 48].

Vocational rehabilitation programs for people with mental disorders, such as supported employment (SE) or its manualized form Individual Placement and Support (IPS), provide comprehensive support in gaining and maintaining competitive employment [49]. SE practitioners provide individualized, long-term job search and retention support integrated with mental health services. SE is an internationally used and effective approach to helping people with a range of mental health conditions to get back to work [50, 51]. With regard to mental health outcomes, however, a recent meta-analysis found heterogeneous, but overall non-significant effects of IPS [52]. In addition, van Rijn et al. [53] reviewed the effect of re-employment programs on different health outcomes and failed to find a significant association with functioning. One explanation for the heterogeneous results is that rather than the intervention alone, taking up competitive employment is the crucial part in improving non-vocational outcomes such as negative symptoms, quality of life and functioning [54]. For instance, Burns et al. [55] found no difference in non-vocational outcomes between the IPS and control group. However, compared to participants who did not work, those who worked showed better global functioning, fewer symptoms and less social disability after 18 months follow-up. Moreover, Jäckel et al. [39] showed that the significant effect of IPS on reduced days of hospitalization and improved quality of life was fully mediated by increased employment rates.

Only a few studies in the SE context included self-assessment measures of functioning or disability, or specifically addressed people with MDD, although it is a highly prevalent illness and leads to many work-related impairments. Furthermore, functioning includes a wide range of domains and it can be helpful to get a more detailed picture of certain functional domains as experienced by individuals with MDD in order to draw appropriate conclusions and offer individual support. The ICF framework provides a structured way to cover many relevant functional domains including participation and is a relevant framework for clinical practice [56]. The most recent version of the diagnostic and statistical manual of mental disorders (DSM-5) [57] suggests using the World Health Organization Disability Assessment Schedule 2 (WHODAS 2.0) to measure functioning and disability based on the ICF “activity and participation” dimensions. The WHODAS 2.0 includes the following six domains: cognition, mobility, self-care, getting along (interacting with other people), life activities (domestic responsibilities, leisure, work & school) and participation. A recent study from Taiwan [58] reported that reemployment compared with continuous unemployment was associated with greater improvement in WHODAS 2.0 scores in people with chronic MDD.

The aim of this study was to examine the relationship between work re-entry and subsequent functioning and depressive symptoms in SE participants with MDD. Based on the existing literature, we hypothesized that re-entering competitive employment would significantly predict better overall functioning and lower symptom severity. An additional aim of this study was to explore whether re-entering work is associated with functional domains based on the ICF participation and activities section and whether participant characteristics, such as age, gender, education and employment status at baseline significantly affect the relationship between work and functioning.

## Method

### Setting & design

This study is a secondary analysis of data collected as part of a larger quasi-experimental trial called IPS-ZIB (English: IPS-Coaching - Back to Working Life), which evaluated an SE intervention at two German sites. IPS-ZIB was preregistered with the German Clinical Trials Register (DRKS; ID: DRKS00023521) and approved by the Ethics Committee of the University of Münster (registration number: 2020-785-b-S) and the Ethics Committee of the University Medicine Greifswald (registration number: BB 162/20). For the current study, we analyzed baseline and two-year follow-up data of participants with depressive disorders in the IPS intervention group. Data were collected between December 2020 and May 2024. The results of the current study are reported in accordance with the STROBE guidelines for reporting observational studies [59].

### Participants

According to the a priori defined sample size, a total of *N* = 150 participants were enrolled in IPS-ZIB to receive the IPS intervention. All participants (*N* = 150) were recruited during or up to three months after psychiatric hospitalization. Other inclusion criteria were a minimum age of 26 years and complex psychosocial support needs, which describe problems in different areas of life based on an instrument used in the regular German rehabilitation system. In accordance with legal requirements for medical rehabilitation services in Germany, all participants had worked for at least six months in the two years prior to study entry. Exclusion criteria were ongoing substance dependence or insufficient knowledge of the German language.

For the purposes of the current study, we excluded participants with a diagnosis other than depressive disorder (*N* = 21) and only analyzed data from participants with a depressive disorder diagnosis (*N* = 129). Diagnoses were obtained from clinical records.

### Procedure

Staff at the psychiatric wards approached potential participants and asked if they were interested in receiving IPS coaching as part of a research study. All participants gave written informed consent and baseline data were collected in a face-to-face appointment lasting approximately 1.5 h. Baseline data included demographics, occupational history and psychosocial questionnaires (see preregistration for details). During the two-year study period, short interim surveys were conducted with participants every six months, either in person or by telephone/mail, to monitor their vocational status. At the end of the study period (two-year follow-up), the comprehensive baseline survey including measures of symptom severity and functioning was repeated in person or by phone and mail if participants were unable to attend an in-person appointment.

During the two-year study period, all participants received a SE intervention guided by the IPS principles [60] by trained and supervised coaches for a maximum of two years. The eight IPS principles include the goal of competitive employment, zero exclusion (i.e., anyone who wants to work can participate), focus on client preferences, rapid job search, integration with mental health care, benefits counseling, systematic job development, and long-term support [60]. A self-assessment of IPS fidelity using the 25-item IPS Fidelity Scale [61] showed overall fair fidelity (88 points).

### Measures

#### Work re-entry

In accordance with established SE outcome measures [62], we used re-entering competitive employment (i.e. returning to work from sick leave or starting a new job) as a grouping variable. Participants were asked every six months whether they had worked competitively during that period. Participants who answered “yes” at any of these time points were classified as employed. In cases where the research team was unable to contact participants, these data were collected using the IPS coaching records to the extent possible. Participants were classified as not working if they dropped out of the study before the end of the two-year observation period and had not worked until that time (*N* = 22).

#### Functioning

Functioning was assessed using the German version of the WHO Disability Assessment Schedule 2.0 36-item interviewer version (WHODAS 2.0) [63], which covers functioning and disability in six domains. For each domain, the interviewer asks: “In the past 30 days, how much difficulty did you have in…”, followed by several items that participants answer on a five point scale. Response categories include 0: none, 1: mild, 2: moderate, 3: severe, 4: extreme or cannot do. The subdomains include:

1. cognition– understanding and communicating (six items, e.g.: “Concentrating on doing something for ten minutes?”).
2. mobility– moving and getting around (five items, e.g. “Standing for long periods such as 30 minutes?”),
3. self-care– hygiene, dressing, eating and staying alone (four items, e.g. “Washing your whole body?”).
4. getting along– interacting with other people (five items, e.g. “Dealing with people you do not know?”).
5. life activities - domestic responsibilities, leisure, work & school (subdomain domestic responsibilities: four items, e.g. “Taking care of your household responsibilities”; subdomain work/school: four items, e.g. “your day-to-day work/school?”).
6. participation– joining in community activities (eight items, e.g. “In the past 30 days: How much of a problem did you have in joining in community activities (for example, festivities, religious or other activities) in the same way as anyone else can?”

In accordance with the manual, the life activity domain was reduced to four items measuring domestic responsibilities, as participants were unable to respond to work or school functioning at baseline. According to the manual, the complex (or “item-response-theory based”) scoring method was applied that takes into account the item difficulty for each item. The scoring follows three steps: “1. Summing of recoded item scores within each domain, 2. Summing of all six domain scores, 3. Converting the summary score into a metric ranging from 0 to 100 (where 0 = no disability; 100 = full disability)” [63]. The scoring was conducted using the syntax provided in the manual resulting in six domain scores and a total score each ranging from 0 to 100 with higher scores indicating more severe disability. The WHODAS 2.0 has demonstrated good psychometric properties and is suitable for use with people with physical and mental disorders [64].

#### Symptom severity

Depressive symptom severity was assessed by the German version [65] of the Beck’s Depression Inventory II (BDI-II) [66]. The BDI-II is a widely used 21-item self-rating questionnaire that measures the severity of depressive symptoms. In each item, participants choose one of four statements that best describes how they felt during the previous two weeks. The statements are scored from zero to three, with higher scores indicating more severe symptoms, and all item scores are added to create a total score. The original and German versions of the BDI-II have good psychometric properties [65, 66].

#### Moderator variables

Moderator variables were measured at baseline and included age (in years), sex, highest education, and current employment status at the time of study entry (current employment contract yes/no). Regardless of having an employment contract, all study participants were not working at the time of study entry (unemployed or on sick leave).

### Statistical analyses

Statistical analyses were conducted using SPSS Statistics Version 29.0. Descriptive statistics are shown for baseline sample characteristics and change in WHODAS 2.0 scores between baseline and follow-up. Baseline differences between participants who worked and did not work were tested using two-sided t-tests and changes in WHODAS 2.0 scores from baseline to follow-up were tested using paired two-sided t-tests. The main analysis was carried out using linear regression analyses with WHODAS 2.0 total score, subdomain scores and BDI-II at two-year follow-up as outcomes. In addition to work re-entry as a predictor, age and the respective baseline values of the outcome were included as covariates to account for baseline differences. In each regression model, covariates were entered into the model first, and work re-entry was added in a second step to determine change in R². Additional models for predicting the follow-up WHODAS 2.0 total score included interaction effects of sex, age, education (dichotomized into university entrance qualification yes/no), and employment contract at baseline with employment in an additional block.

#### Missing data

Complete data at baseline and two-year follow-up in the WHODAS 2.0 and subdomains were available from 62 participants (48.1%) and complete BDI-II data were available from 79 (61.2%) participants. Baseline data were mostly complete, with missing values for each WHODAS 2.0 domain ranging from 0 to 13% and for the BDI-II 7.0%. At baseline, values were missing only at item level, with most missing values in domain four due to one item asking about problems with sexual activities. Missing values in the outcome variables (two-year follow-up) were mainly caused by loss to follow-up so that the WHODAS 2.0 and BDI-II were missing completely (33-38%). The main reason for loss to follow-up was that participants could no longer be reached. All available measures collected in the framework of the original study (see preregistration for details) were used to analyze and handle missing values. Participants who did not work and who reported worse baseline depressive symptoms (BDI-II), cognitive deficits (questionnaire for complaints of cognitive disturbances: FLei) [67], and mental health (12-item short form health survey: SF-12) [68] were more likely to have missing data at follow-up.

Multiple imputation was used as the state of the art method to handle missing data, because there were other variables in the data set that were related to the missingness or the WHODAS 2.0/BDI-II values and the missing mechanism was assumed to be missing at random (MAR) [69]. We created 30 multiply imputed data sets under fully conditional specification using predictive mean matching with number of donors set to 5 (default). Because of the high number of items and the small sample size, we used parcel summary imputation to impute each WHODAS domain individually and BDI-II at baseline and follow-up on the item level [70]. Parcel summary imputation is a method to reduce the amount of variables in the imputation model by using temporary average scores over the available items (“parcel summary scores”) as predictors for the imputation of items from other scales [70]. We used the temporary mean scores of the WHODAS 2.0 domains and the BDI-II as predictors and merged the imputed datasets as suggested. In addition, we included the interaction terms used in the analysis models and auxiliary baseline variables that were related either to WHODAS 2.0 or BDI-II scores or to loss to follow-up into the imputation models. Imputed values were found to be comparable to observed values and the main results are reported according to the fully imputed models. In addition, results of the complete case analyses are reported in additional file 1 and any differences in statistical significance decisions between results of complete case analyses and fully imputed models are reported in the main text. All reported estimates of fully imputed data are based on pooled results provided by SPSS when running linear regression analysis using a multiple imputation dataset. Pooled standard deviations and R-squared statistics were calculated using the mean across all imputed datasets [71].

## Results

### Baseline characteristics

Participants (*N* = 129) were on average 42 years old (*SD* = 11 years) and 54.3% (*N* = 70) were female. At baseline, *N* = 83 participants (64.3%) held an employment contract and 51 (39.5%) had a higher education (i.e. university entrance qualification). Table 1 shows the descriptive sample characteristics at baseline and follow-up separately for participants with a work re-entry and without a work re-entry during the two-year study period. In total, *N* = 77 participants (59.7%) worked in a competitive job during the two-year observation period. Of these, 24 participants (31.2%) returned to an old job and 53 (68.8%) started a new job. On average, participants started work after *Md* = 164 days (5.4 months). Compared to participants who did not work, participants who worked were significantly younger, *t*(127) = 2.167, *p* =.030, and had less impairment at baseline in domains two (mobility), *t*(127) = 2.658, *p* =.008, and five (domestic responsibilities), *t*(127) = 2.381, *p* =.017, as well as in the WHODAS 2.0 total score, *t*(127) = 2.079, *p* =.038. Descriptively, most severe impairment at baseline was present in domain six (participation), followed by domains five (domestic responsibilities), four (interacting with others) and one (cognitive functioning). Less severe impairment was observed in domains two (mobility) and three (self-care).

**Table 1:** Descriptive functional and symptom severity scores at baseline and follow-up for imputed data (*N* = 129)

	Baseline						Follow-up					
	Total sample		Work re-entry				Total sample		Work re-entry			
	*N* = 129		Yes (*N* = 77)		No (*N* = 52)		*N* = 129		Yes (*N* = 77)		No (*N* = 52)	
	***M (SD)***		***M (SD)***		***M (SD)***		***M (SD)***		***M (SD)***		***M (SD)***	
**WHODAS 2.0 total**	45.91 (15.91)		43.54 (15.77)		49.40 (15.61)		30.53 (17.49)**		25.43 (15.28)**		38.09 (17.93)**	
**WHODAS 2.0 domain 1** Cognition– understanding & communicating	47.79 (19.18)		45.95 (18.76)		50.51 (19.65)		31.39 (20.85)**		25.13 (17.34)**		40.65 (22.20)*	
**WHODAS 2.0 domain 2** Mobility– moving & getting around	28.15 (25.40)		23.38 (22.91)		35.22 (27.43)		22.12 (23.64)*		16.48 (19.33)*		30.48 (26.90)	
**WHODAS 2.0 domain 3** Self-care– hygiene, dressing, eating & staying alone	17.05 (17.91)		14.81 (16.19)		20.38 (19.90)		12.45 (15.77)*		9.31 (14.01)*		17.11 (17.09)	
**WHODAS 2.0 domain 4** Getting along– interacting with other people	51.37 (26.06)		49.62 (26.73)		53.96 (25.07)		37.75 (25.78)**		33.79 (25.87)**		43.62 (24.67)*	
**WHODAS 2.0 domain 5.1** Life activities– domestic responsibilities	55.34 (27.74)		50.65 (26.87)		62.29 (27.80)		35.89 (26.00)**		32.26 (23.58)**		41.26 (28.52)**	
**WHODAS 2.0 domain 6** Participation– joining in community activities	61.53 (18.30)		60.95 (19.62)		62.38 (16.29)		37.12 (21.61)**		31.33 (19.85)**		45.68 (21.38)**	
**BDI-II** Symptom severity	25.63 (11.28)		24.16 (9.74)		27.81 (13.03)		15.36 (12.89)**		12.85 (11.54)**		19.09 (13.89)*	

*Notes. M* = mean; *SD* = standard deviation; *p* values based on two-tailed paired t-tests baseline to follow-up; *p <.05; **p <.001

### Change from baseline to follow-up

As shown in Table 1, on average participants significantly improved in the overall WHODAS 2.0 score as well as in all functional domains and symptom severity from baseline to two-year follow-up. Statistically significant improvements were observed in both subgroups with the exception of mobility (*p* =.234) and self-care (*p* =.361) in the group who did not work. Across both employment groups, domains four (interacting with others), five (household responsibilities), six (participation) and one (cognition) had the highest average scores at follow-up. Similar to baseline measures, the lowest impairment was observed in domain three (self-care), followed by domain two (mobility).

In contrast to the results of the imputed models, the complete case analysis did not show statistically significant improvement in mobility (*p* =.150) and marginal significance in self-care (*p* =.050).

### Work and functioning

Table 2 shows the results of the main regression analyses testing the relationship between work re-entry and the outcomes variables at follow-up. Adjusted for baseline functioning and age, participants who worked during the two-year observation period had significantly lower overall WHODAS 2.0 at follow-up than participants who did not work. Specifically, if participants had worked, the WHODAS 2.0 total score decreased on average by 8.95 points, which relates to improved overall functioning. In terms of functional domains, work re-entry was significantly associated with less impairment at follow-up in the cognition and participation domains. Regarding symptom severity (*p* =.054) and mobility (*p* =.072), the results suggest a marginally nonsignificant association of work re-entry with better scores. With the exception of symptom severity (BDI-II), for which the complete case analysis suggested a statistically significant association with return to work (*p* =.003), the results of the complete case analysis did not differ in the significance decision (see additional file 1). Additional file 2 shows the results of the regression models unadjusted for age.

**Table 2:** Results of imputed linear regression models predicting functioning (WHODAS 2.0 total and domain scores) and symptom severity (BDI-II total score) (*N* = 129)

	b [95% CI]	SE	T	*p*	*R*²	Change in *R*²
**T2 WHODAS 2.0 total**						
Age	0.16 [-0.08; 0.41]	0.13	1.30	0.194		
T1 WHODAS 2.0 total	0.52 [0.35; 0.68]	0.09	6.08	< 0.001	0.305	
Work re-entry	-8.95 [-14.48; -3.42]	2.82	-3.17	0.002	0.366	0.060
**T2 WHODAS 2.0 domain 1** Cognition– understanding & communicating						
Age	0.17 [-0.22; 0.55]	0.19	0.85	0.396		
T1 WHODAS 2.0 domain 1	0.31 [0.12; 0.49]	0.10	3.25	0.001	0.135	
Work re-entry	-13.43 [-21.58; -5.28]	4.14	-3.25	0.001	0.235	0.100
**T2 WHODAS 2.0 domain 2** Mobility– moving & getting around						
Age	0.47 [0.05; 0.89]	0.21	2.20	0.029		
T1 WHODAS 2.0 domain 2	0.38 [0.22; 0.54]	0.08	4.67	< 0.001	0.310	
Work re-entry	-7.51 [-15.70; -0.68]	4.17	-1.80	0.072	0.334	0.024
**T2 WHODAS 2.0 domain 3** Self-care– hygiene, dressing, eating & staying alone						
Age	0.15 [-0.15; 0.46]	0.16	0.99	0.323		
T1 WHODAS 2.0 domain 3	0.29 [0.10; 0.49]	0.10	2.94	0.004	0.163	
Work re-entry	-5.52 [-12.16; 1.12]	3.37	-1.64	0.103	0.193	0.030
**T2 WHODAS 2.0 domain 4** Getting along– interacting with other people						
Age	0.18 [-0.22; 0.58]	0.20	0.90	0.367		
T1 WHODAS 2.0 domain 4	0.47 [0.30; 0.64]	0.09	5.39	< 0.001	0.255	
Work re-entry	-7.04 [-16.44; 2.36]	4.78	-1.47	0.142	0.274	0.019
**T2 WHODAS 2.0 domain 5.1** Life activities– domestic responsibilities						
Age	-0.03 [-0.48; 0.42]	0.23	-0.13	0.895		
T1 WHODAS 2.0 domain 5.1	0.45 [0.27; 0.62]	0.09	4.98	< 0.001	0.243	
Work re-entry	-3.94 [-14.13; 6.25]	5.18	-0.76	0.447	0.251	0.008
**T2 WHODAS 2.0 domain 6** Participation– joining in community activities						
Age	0.15 [-0.20; 0.51]	0.18	0.84	0.401		
T1 WHODAS 2.0 domain 6	0.41 [0.21; 0.61]	0.10	3.97	< 0.001	0.147	
Work re-entry	-13.12 [-21.42; -4.83]	4.22	-3.11	0.002	0.235	0.088
**T2 BDI-II total** Symptom severity						
Age	0.03 [-0.20; 0.26]	0.12	0.25	0.800		
T1 BDI-II	0.26 [0.03; 0.49]	0.12	2.22	0.028	0.077	
Work re-entry	-5.17 [-10.42; -0.09]	2.67	-1.93	0.054	0.117	0.039

*Notes.* Work re-entry reference category: 0; b: unstandardized regression coefficient, CI: confidence interval; SE: standard error; change in R²: change in R² when work re-entry is included in the model containing the covariates of age and baseline score for each outcome

### Moderators

No significant interaction effects were found between work re-entry and gender (b = -1.83, SE = 5.59, *p* =.743), work re-entry and education (b = -6.14, SE = 6.32, *p* =.331) or work re-entry and employment status at baseline (b = -3.79, SE = 5.68, *p* =.505) predicting overall functioning. However, there was a significant interaction effect between work re-entry and age predicting the WHODAS 2.0 total score at follow-up, b = -0.78, SE = 0.24, *p* =.001, controlled for baseline functioning. Specifically, the relationship between work re-entry and a decreased WHODAS 2.0 total score was stronger the older participants were.

## Discussion

This study aimed to examine the associations between work re-entry and subsequent functioning and symptom severity among participants with MDD in a two-year SE program. The results showed that work re-entry was statistically significantly associated with better overall functioning, cognitive functioning and community participation, after adjusting for baseline scores and age. In addition, the association between work and overall functioning at follow-up was stronger the older the participants were. After adjustment for baseline functioning and age, work re-entry was not significantly associated with self-care, social interaction and household responsibilities, and marginally nonsignificant associations were observed with improved mobility and lower symptom severity.

### Work re-entry and overall functioning

Approximately 60% of the study participants worked in a competitive job during the observational period of two years. Although baseline scores as covariates accounted for significant amounts of variance in follow-up functioning, as hypothesized, work re-entry still made statistically significant contributions to predicting overall functioning. These findings suggest that regardless of the functional impairment with which participants entered the SE intervention, those who entered work were less overall impaired at follow-up. The results support the assumption that work re-entry with a SE program does not cause harm to people with depression (e.g. due to work-related stress) in terms of functioning in a wide range of domains and symptom severity [72]. Moreover, the findings are in line with a study from Taiwan that found a significant relationship between becoming employed and improved functioning according to the WHODAS 2.0 in individuals with chronic MDD [58]. They are also consistent with studies that have found significant associations between entering employment and various improved mental health outcomes in people with mental disorders [35, 37, 38, 41, 55, 73]. Beyond symptomatic improvement, our findings showed that in participants with MDD better self-assessed functioning was also associated with work participation.

There are different interpretations of the association of work re-entry with lower disability. Although we controlled for baseline scores and age, participants who experienced more severe functional impairment during the two years of SE may have been less likely to work [19, 33, 76]. Simultaneously, based on cross-sectional and prospective studies, authors have concluded that under beneficial conditions work can promote mental health and contribute to recovery [29, 35, 77]. Given that all participants in the present study received comprehensive support from SE practitioners, they had support in creating favorable working conditions, suggesting that they may have been able to benefit from the advantages of being employed.

The significant interaction effect between age and work re-entry suggests that work re-entry was associated with better follow-up functioning especially among older participants. While there was little difference in functioning between the working and non-working groups for younger participants, older non-working participants had significantly worse functioning compared to those who worked. This finding may be surprising, given that it has been found that younger men were particularly affected by negative mental health effects of unemployment [26]. However, results regarding the moderating impact of age on the association between (un)employment and mental health are heterogeneous and some have also suggested that middle aged persons were less affected [28, 74]. In particular, in our sample, due to the inclusion criteria, the youngest participants were in their mid-twenties and all had worked in the past, so they may not be comparable to other ‘young’ subsamples aged under twenty. Further, in a large German sample, older men’s health was more affected by job loss than younger men’s [75], which the authors suggest may reflect a stronger identification of older men with the role of worker and provider. However, the cited studies analyzed job loss instead of re-entering work and used large general population samples, so the results are not fully comparable. An additional interpretation could be that older participants had spent more time in the workforce prior to study entry, which may contribute to a stronger interdependence between the ability to work and other functional domains. For example, this could mean that older individuals return to work as soon as they feel better. Future studies of SE samples may examine the association between re-entering work and functioning in different age groups to understand this relationship more deeply and draw conclusions for SE programs.

### Functional domains

Cognitive impairment, which was the domain most strongly associated with work, is seen as a core feature of MDD and experienced deficits have been found to persist even in people with remitting MDD [78, 79]. Cognition is of course relevant to the performance of work-related tasks and has been associated with daily functioning [80, 81, 82, 83] and better employment outcomes [84, 85] in people with depression. Thus, one explanation for the present finding is that individuals who experience more persistent cognitive deficits over the course of the two-year follow-up may be less likely to work. Another possibility is that the self-assessed cognitive deficits can be explained by a negative bias due to depressive symptoms. Since depressive symptom severity improved over the course of the study, it may also be that a decrease in negatively biased evaluation of cognitive functioning explain the improved self-assessment [86]. In addition, successfully completing tasks at work could strengthen confidence in the own cognitive capacities and promote self-efficacy and subjectively improved cognition [87]. However, given that both groups reported some cognitive disability at follow-up, certain individuals in SE may profit from additional support in terms of cognitive symptoms or from adjustment of work conditions.

Alongside cognition, participation was the other domain most strongly associated with re-entering work, with work adding approximately 9% of explained variance in follow-up functioning. The participation domain includes eight items measuring community participation in social events, being able to perform leisure activities, experienced barriers to participation by others, or the impact of a health condition on family life. Many benefits of being employed that have been reported correspond with the participation domain. Qualitative studies have shown that people with mental disorders associate gaining employment with more positive self-appraisal, greater optimism, remuneration and autonomy, status and acceptance in society, a sense of purpose and feeling useful to others, and improved relationships [88]. These benefits may explain improved self-reported participation in several ways. First, participating in community activities can at least in part depend on financial resources, with one item even asking for financial burden due to the health condition. Second, as competitive work is socially desirable in Western societies such as Germany, the self-assessed participation in society might depend to some degree on identifying as a worker and contributing to society by being employed [89, 90, 91, 92]. For instance, participants in a study by Koletsi et al. [93] described work as helpful to get “’acceptance’, a ‘purpose in life’, a ‘sense of belonging’ and ‘re-integration into society” (p. 967, second paragraph). Furthermore, being employed promotes social contacts, which provides more opportunities to participate in social events [93]. In sum, the relationship between work and a broader societal participation stresses the important role of vocational rehabilitation interventions in promoting work participation. Given the significant impairment in work participation, it is also important in the clinical treatment context to address work-related needs and provide support regarding vocational rehabilitation [94].

Other functional domains, such as household responsibilities, may be activities more distant from work than cognition or participation, which could explain the non- or marginally statistically significant results and smaller effect sizes. With regard to self-care and mobility, there may be floor effects, because our sample already had relatively low disability scores at baseline. Because the interaction domain had most missing values, results in this domain should also be interpreted with caution. While the complete case analysis supported the associations observed between work re-entry and overall functioning, participation and cognition, it would additionally suggest a significant association between work re-entry and symptom severity. Because missing values depended on the employment group, the complete case analysis may have produced biased results and in sum, we decided to focus on the results of the imputed analyses.

### Development from baseline to follow-up

Consistent with previous research, we observed overall significant improvements in all functional domains and symptom severity, but still some impairment among the study participants at follow-up [12, 16]. Compared to complete case analysis, the larger sample size in the imputed models led to additional statistically significant improved scores of mobility and self-care. While participants on average had moderately severe depressive symptoms at baseline, they had on average mild depressive symptoms at follow-up [65]. However, participants in both the work re-entry and no-work group still reported some disability at follow-up, particularly in interacting with others, participating in society, household activities, and cognitive functioning. This is in line with a recent study that found residual disability even in participants with remitted depression compared to healthy controls [12]. In addition, consistent with our findings, they found that the domains of mobility and self-care were the least impaired, while more disability was present in the domains of household tasks, interaction with others, community participation, and cognition. Baseline differences in overall functioning, mobility, and domestic responsibilities between those who entered the workforce and those who did not suggest that returning to work may have been more difficult with greater impairment in these basic capacities. In general, the SE intervention all participants received aims to include everyone who expresses a desire to work and research has shown that SE can be successful regardless of client characteristics [95]. However, some participants may need more time and return to work at a later point due to capacity limitations in basic areas of daily living.

### Limitations

As already mentioned, there was a high amount of missing data, especially at follow-up and although we used multiple imputation as the state of the art method, we cannot exclude bias and as described, there were differences between the complete case analyses and analyses of the imputed models regarding symptom severity. Furthermore, due to the observational study design, it is possible that confounders not measured in the present study affected the results. It should also be noted that no information was recorded on how many people were initially approached for recruitment to the study. Another point to consider it that we only used a dichotomous measure of whether or not participants worked in a competitive job at some point during the observation period. Although this is a widely used main outcome measure in SE research and does represent an important “step” in vocational rehabilitation, it remains unclear whether participants held the job until follow-up and whether other measures, such as the duration of employment, would have led to other results. In addition, it was not within the scope of this study to assess workplace functioning, so that it remains unclear how those participants who entered work experienced their work functioning. While the WHODAS 2.0 is an internationally used and detailed measure of functioning, there is no “minimal clinically important difference” [96], which limits the interpretation of the WHODAS 2.0 results. In addition, although the WHODAS 2.0 was designed to measure the ICF activity and participation levels, it also includes symptoms (cognitive functioning) and does not allow a clear conceptual distinction between the levels. In future studies, a distinct measurement of the three levels (symptoms, activities/capacities, and participation) may allow for a more precise interpretation of the association between symptoms, activity and participation limitations. Instruments that specifically target one of these levels include for example the Mini-ICF-APP, which measures activity impairment [97]. Finally, the results are limited to the current sample of participants with depressive disorders that were selected by medical staff to receive a SE intervention within the German welfare system. Future studies have to compare the effects of re-employment on symptomatic and functional remission in different settings of vocational rehabilitation.

## Conclusions

The results of the present study suggest that re-entering work was associated with better subsequent self-reported functioning in people with MDD who participated in an SE intervention. Using the ICF framework of functioning, cognition and community participation were the functional domains most strongly associated with work re-entry. Furthermore, the relationship between work re-entry and overall functioning was stronger in older participants. The findings support previous research on the association between entering work and improved mental health related outcomes and suggest that this association seems to apply in a sample of MDD patients participating in an SE program in Germany. However, it may be important to examine functional domains independently and to stratify by age group. The association between re-entering work and other key domains of functioning emphasizes the important contribution of vocational rehabilitation interventions in promoting competitive employment among people with MDD. Functioning in daily life activities other than work may play a role in these interventions, possibly both in terms of persistent functional impairment as a potential barrier to employment and in terms of the benefits of working in good conditions. For clinical practice, it is important to address clients’ needs regarding work participation and functional recovery.

## Electronic supplementary material

Below is the link to the electronic supplementary material.

Supplementary Material 1: Additional_file1.docx; additional tables 3 and 4 show the results of the data analysis when only complete cases are included.

Supplementary Material 2: Additional_file2.docx; additional table 5 shows the results of the imputed linear regression models unadjusted for age.

## Data Availability

The datasets analyzed during the current study are not publicly available due to their containing information that could compromise the privacy of research participants, but are available from the corresponding author on reasonable request.

## Abbreviations

MDD: Major depressive disorder
ICF: International Classification of Functioning, Disability and Health
SE: Supported employment
IPS: Individual placement and support
DSM-5: Diagnostic and Statistical Manual of Mental Disorders Fifth Edition
WHODAS 2.0: WHO Disability Assessment Schedule 2.0
BDI-II: Beck’s Depression Inventory II
SD: Standard deviation
SE: standard error
CI: Confidence interval
