# Supplementary material for: Work re-entry and functioning in people with major depression: a longitudinal study of supported employment participants
Source: BMC Psychiatry. 2025 Apr 18;25:402. doi: 10.1186/s12888-025-06826-z (PMC12008965; doi:10.1186/s12888-025-06826-z)
Supplement: Supplementary file 1 — Supplementary Material 1: Additional_file1.docx; additional tables 3 and 4 show the results of the data analysis when only complete cases are included. [file 12888_2025_6826_MOESM1_ESM.docx]

|  | **Baseline** | | | | | | | | |  | **Follow-up** | | | | | | |  |
| --- | --- | --- | --- | --- | --- | --- | --- | --- | --- | --- | --- | --- | --- | --- | --- | --- | --- | --- |
|  | **Total sample** | |  | **Work re-entry** | | | | |  | **Total sample** | |  | **Work re-entry** | | | | | |
|  | **N = 129** | |  | **Yes (N = 77)** | |  | **No (N = 52)** | |  | **N = 129** | |  | **Yes (N = 77)** | |  | **No (N = 52)** | |  |
|  | **N** | ***M (SD)*** |  | **N** | ***M (SD)*** |  | **N** | ***M (SD)*** |  | **N** | **M (SD)** |  | **N** | ***M (SD)*** |  | **N** | ***M (SD)*** |  |
| **WHODAS 2.0 total** | 104 | 45.50 (16.15) |  | 62 | 42.18 (15.22) |  | 42 | 50.39 (16.41) |  | 79 | 28.43 (20.22)** |  | 59 | 23.58 (16.30)** |  | 20 | 42.72 (24.10) |  |
| **WHODAS 2.0 domain 1**  Cognition – understanding & communicating | 125 | 47.88 (19.15) |  | 75 | 46.13 (18.59) |  | 50 | 50.50 (19.85) |  | 86 | 30.23 (22.89) ** |  | 63 | 24.21 (17.94)** |  | 23 | 46.74 (26.99) |  |
| **WHODAS 2.0 domain 2**  Mobility– moving & getting around | 129 | 28.15 (25.40) |  | 77 | 23.38 (22.91) |  | 52 | 35.22 (27.43) |  | 87 | 21.77 (25.47) |  | 63 | 15.87 (19.85) |  | 24 | 37.24 (31.89) |  |
| **WHODAS 2.0 domain 3**  Self-care– hygiene, dressing, eating & staying alone | 129 | 17.05 (17.92) |  | 77 | 14.81 (16.19) |  | 52 | 20.38 (19.90) |  | 87 | 11.61 (16.20) |  | 63 | 9.37 (14.69)* |  | 24 | 17.50 (18.71) |  |
| **WHODAS 2.0 domain 4**  Getting along– interacting with other people | 112 | 50.30 (26.59) |  | 66 | 46.97 (26.98) |  | 46 | 55.07 (25.55) |  | 80 | 35.93 (28.30)** |  | 60 | 31.67 (26.30)** |  | 20 | 48.75 (30.86) |  |
| **WHODAS 2.0 domain 5.1**  Life activities– domestic responsibilities | 127 | 55.28 (27.71) |  | 77 | 50.65 (26.87) |  | 50 | 62.40 (27.74) |  | 87 | 35.06 (27.82)** |  | 63 | 30.79 (24.12)** |  | 24 | 46.25 (33.86)* |  |
| **WHODAS 2.0 domain 6**  Participation– joining in community activities | 123 | 61.31 (18.46) |  | 73 | 60.39 (19.79) |  | 50 | 62.67 (16.43) |  | 85 | 35.83 (24.17)** |  | 62 | 29.84 (20.75)** |  | 23 | 51.99 (25.74) |  |
| **BDI-II**  Symptom severity | 120 | 25.63 (11.28) |  | 70 | 24.39 (9.59) |  | 50 | 28.14 (13.04) |  | 83 | 14.57 (12.44)** |  | 62 | 12.26 (11.17)** |  | 21 | 21.38 (13.75) |  |

**Additional file 1.**

**Title:** Additional tables 3 and 4

**Description:** Results of the data analysis when only complete cases are included

*Table 3*. Descriptive functional and symptom severity scores at baseline and follow-up for complete cases

*Notes. M* = mean; *SD* = standard deviation; *p* values based on two-tailed paired t-tests baseline to follow-up; **p* < .05; ***p* < .001.

*Table 4*. Linear regression results of the complete case analysis

|  |  | **N** | **b**  **[95 % CI]** | **SE** | **T** | ***p*** | **R²** | **Change in R²** |
| --- | --- | --- | --- | --- | --- | --- | --- | --- |
| **T2 WHODAS 2.0 total** |  | 62 |  |  |  |  |  |  |
| Age |  |  | 0.23 [-0.18; 0.64] | 0.21 | 1.10 | .275 |  |  |
| T1 WHODAS 2.0 total |  |  | 0.64 [0.38; 0.90] | 0.13 | 4.87 | <.001 | .381 |  |
| Work re-entry |  |  | -15.22 [-24.77; -5.66] | 4.77 | -3.19 | .002 | .473 | .092 |
| **T2 WHODAS 2.0 domain 1**  Cognition – understanding & communicating |  | 83 |  |  |  |  |  |  |
| Age |  |  | 0.08 [-0.34; 0.50] | 0.21 | 0.37 | .716 |  |  |
| T1 WHODAS 2.0 domain 1 |  |  | 0.40 [0.17; 0.62] | 0.12 | 3.45 | .001 | .178 |  |
| Work re-entry |  |  | -19.77 [-30.00; -9.55] | 5.14 | -3.85 | <.001 | .308 | .130 |
| **T2 WHODAS 2.0 domain 2**  Mobility– moving & getting around |  | 87 |  |  |  |  |  |  |
| Age |  |  | 0.40 [-0.02; 0.82] | 0.21 | 1.90 | .061 |  |  |
| T1 WHODAS 2.0 domain 2 |  |  | 0.50 [0.31; 0.69] | 0.10 | 5.29 | <.001 | .387 |  |
| Work re-entry |  |  | -9.86 [-20.07; 0.36] | 5.14 | -1.92 | .058 | .413 | .026 |
| **T2 WHODAS 2.0 domain 3**  Self-care– hygiene, dressing, eating & staying alone |  | 87 |  |  |  |  |  |  |
| Age |  |  | 0.16 [-0.14; 0.46] | 0.15 | 1.07 | .286 |  |  |
| T1 WHODAS 2.0 domain 3 |  |  | 0.41 [0.21; 0.61] | 0.10 | 4.12 | <.001 | .203 |  |
| Work re-entry |  |  | -6.75 [-13.91; 0.41] | 3.60 | -1.88 | .064 | .236 | .032 |
| **T2 WHODAS 2.0 domain 4**  Getting along– interacting with other people |  | 69 |  |  |  |  |  |  |
| Age |  |  | 0.32 [-0.28; 0.91] | 0.30 | 1.06 | .295 |  |  |
| T1 WHODAS 2.0 domain 4 |  |  | 0.59 [0.36; 0.82] | 0.12 | 5.11 | <.001 | .326 |  |
| Work re-entry |  |  | -13.36 [-26.91; 0.18] | 6.78 | -1.97 | .053 | .364 | .038 |
| **T2 WHODAS 2.0 domain 5.1**  Life activities– domestic responsibilities |  | 86 |  |  |  |  |  |  |
| Age |  |  | -0.08 [-0.56; 0.41] | 0.25 | -0.31 | .756 |  |  |
| T1 WHODAS 2.0 domain 5.1 |  |  | 0.55 [0.36; 0.75] | 0.10 | 5.62 | <.001 | .306 |  |
| Work re-entry |  |  | -9.55 [-21.47; 2.38] | 6.00 | -1.59 | .115 | .327 | .021 |
| **T2 WHODAS 2.0 domain 6**  Participation– joining in community activities |  | 79 |  |  |  |  |  |  |
| Age |  |  | 0.13 [-0.31; 0.57] | 0.22 | 0.60 | .551 |  |  |
| T1 WHODAS 2.0 domain 6 |  |  | 0.52 [0.28; 0.76] | 0.12 | 4.28 | <.001 | .202 |  |
| Work re-entry |  |  | -21.77 [-32.49; -11.04] | 5.38 | -4.04 | <.001 | .345 | .143 |
| **T2 BDI-II**  Symptom severity |  | 79 |  |  |  |  |  |  |
| Age |  |  | -0.01 [-0.25; 0.24] | 0.12 | -0.05 | .963 |  |  |
| T1 BDI-II |  |  | 0.45 [0.19; 0.71] | 0.13 | 3.43 | .001 | .140 |  |
| Work re-entry |  |  | -9.05 [-14.99; -3.12] | 2.98 | -3.04 | .003 | .234 | .094 |

*Notes.* Work re-entry reference category: 0; b: unstandardized regression coefficient, CI: confidence interval; SE: standard error; change in R²: change in R² when entering work re-entry into the model with the covariates age and baseline score of the respective outcome.
