# Supplementary material for: Work re-entry and functioning in people with major depression: a longitudinal study of supported employment participants
Source: BMC Psychiatry. 2025 Apr 18;25:402. doi: 10.1186/s12888-025-06826-z (PMC12008965; doi:10.1186/s12888-025-06826-z)
Supplement: Supplementary file 2 — Supplementary Material 2: Additional_file2.docx; additional table 5 shows the results of the imputed linear regression models unadjusted for age. [file 12888_2025_6826_MOESM2_ESM.docx]

**Additional file 2.**

**Title:** Additional table 5

**Description:** Results of imputed linear regression models unadjusted for age

*Table 5*. Results of imputed linear regression models predicting functioning (WHODAS 2.0 total and domain scores) and symptom severity (BDI-II total score) unadjusted for age (N = 129)

|  | **b**  **[95% CI]** | **SE** | **T** | ***p*** | **R²/change in R²** |
| --- | --- | --- | --- | --- | --- |
| **T2 WHODAS 2.0 total** |  |  |  |  | .355 |
| T1 WHODAS 2.0 total | 0.53 [0.37; 0.70] | 0.08 | 6.34 | <.001 | .285 |
| Work re-entry | -9.54 [-14,98; -4.09] | 2.78 | -3.44 | <.001 | .070 |
| **T2 WHODAS 2.0 domain 1**  Cognition – understanding & communicating |  |  |  |  | .224 |
| T1 WHODAS 2.0 domain 1 | 0.32 [0.14; 0.51] | 0.10 | 3.38 | <.001 | .087 |
| Work re-entry | -14.06 [-21.85; -6.27] | 3.96 | -3.55 | <.001 | .111 |
| **T2 WHODAS 2.0 domain 2**  Mobility– moving & getting around |  |  |  |  | .288 |
| T1 WHODAS 2.0 domain 2 | 0.43 [0.27; 0.59] | 0.08 | 5.26 | <.001 | .202 |
| Work re-entry | -8.93 [-17.19; -0.66] | 4.21 | -2.12 | .034 | .034 |
| **T2 WHODAS 2.0 domain 3**  Self-care– hygiene, dressing, eating & staying alone |  |  |  |  | .177 |
| T1 WHODAS 2.0 domain 3 | 0.30 [0.10; 0.49] | 0.10 | 3.04 | .003 | .116 |
| Work re-entry | -6.13 [-12.53; 0.26] | 3.25 | -1.89 | .060 | .037 |
| **T2 WHODAS 2.0 domain 4**  Getting along– interacting with other people |  |  |  |  | .267 |
| T1 WHODAS 2.0 domain 4 | 0.47 [0.30; 0.64] | 0.09 | 5.48 | <.001 | .230 |
| Work re-entry | -7.78 [-16.88; 1.33] | 4.63 | -1.68 | .094 | .024 |
| **T2 WHODAS 2.0 domain 5.1**  Life activities– domestic responsibilities |  |  |  |  | .248 |
| T1 WHODAS 2.0 domain 5.1 | 0.44 [0.27; 0.62] | 0.09 | 5.02 | <.001 | .216 |
| Work re-entry | -3.83 [-13.70; 6.04] | 5.02 | -0.76 | .445 | .008 |
| **T2 WHODAS 2.0 domain 6**  Participation– joining in community activities |  |  |  |  | .227 |
| T1 WHODAS 2.0 domain 6 | 0.41 [0.20; 0.61] | 0.10 | 3.94 | <.001 | .119 |
| Work re-entry | -13.76 [-21.83; -5.70] | 4.10 | -3.36 | <.001 | .100 |
| **T2 BDI-II total**  Symptom severity |  |  |  |  | .114 |
| T1 BDI-II | 0.26 [0.04; 0.49] | 0.12 | 2.27 | .024 | .055 |
| Work re-entry | -5.28 [-10.46; -0.10] | 2.64 | -2.01 | .046 | .042 |

*Notes.* Work re-entry reference category: 0; b: unstandardized regression coefficient, CI: confidence interval; SE: standard error; R² for each predictor corresponds to change in R² when entering the predictor into the model.
